# Switch of macrophage fusion competency by 3D matrices

**DOI:** 10.1038/s41598-020-67056-9

**Published:** 2020-06-25

**Authors:** Josephine Y. Fang, Zhi Yang, Bo Han

**Affiliations:** 1 Nimni-Cordoba Tissue Engineering and Drug Discovery Laboratory, Division of Plastic and Reconstructive Surgery, Departments of Surgery, Keck School of Medicine, University of Southern California, Los Angeles, California United States; 2 Center of Craniofacial Biology, Herman Ostrow School of Dentistry, University of Southern California, Los Angeles, California United States; 3 Department of Biomedical Engineering, Viterbi School of Engineering, University of Souther California, Los Angeles, California United States

**Keywords:** Cell biology, Immunology

## Abstract

Foreign body reaction reflects the integration between biomaterials and host cells. At the implantation microenvironment, macrophages usually fuse into multinuclear cells, also known as foreign body giant cells, to respond to the biomaterial implants. To understand the biomaterial-induced macrophage fusion, we examined whether biomaterial alone can initiate and control the fusion rate without exogenous cytokines and chemicals. We introduced a collagen-based 3D matrix to embed Raw264.7 cell line and primary rat bone marrow-derived macrophages. We found the biomaterial-stimuli interacted regional macrophages and altered the overall fusogenic protein expressions to regulate the macrophage fusion rate. The fusion rate could be altered by modulating the cell-matrix and cell-cell adhesions. The fused macrophage morphologies, the nuclei number in the fused macrophage, and the fusion rates were matrix dependent. The phenomena were also observed in the *in vivo* models. These results suggest that the biomaterial-derived stimuli exert similar functions as cytokines to alter the competency of macrophage fusion as well as their drug sensitivity in the biomaterial implanted tissue environment. Furthermore, this *in vitro* 3D-matrix model has the potential to serve as a toolbox to predict the host tissue response on implanted biomaterials.

## Introduction

Macrophages are the main players in the foreign body reaction. Their cellular activities are responsible for the destruction and integration of biomaterials. Three critical macrophage responses have been identified in host-material interactions: adhesion, activation, and fusion^1^. Macrophages perform adhesion on the material surfaces via adhesion ligand-receptor interactions. Due to biomaterial physiochemical properties (i.e. material topologies, charges, hydrophilicity/hydrophobicity, stiffness, crosslinking reagents, by-products), macrophages express various types of integrins and complement receptors for adhesion or opsonization^2^. These cell-material interactions can trigger intracellular signals to promote macrophage activation. The adherent macrophages perform activation and fusion to form different subtypes of macrophages or multinucleated cells (MNC). MNC cells are the most distinctive features surrounding biomaterials and are considered to perform more enhanced functions than macrophages^3^. Different from the MNCs observed in the skeletal muscle, placentas, and bones in certain healthy tissues^4,5^, they are part of the innate immune system to battle with foreign pathogens and intrusive materials. Similar MNCs are also observed in the pathological lesions such tuberculosis, atherosclerosis, and cancer.

In current macrophage fusion machinery, macrophages require cytokines (IL-4, IL-13, and RANKL) or chemicals (α-tocopherol, 1α,25-dihydroxyvitamin D3, and 12-ο-tetradecanoyl-phorbol-13-acetate) as fusogenic stimuli^6^. These stimuli convert macrophage into fusion-competent macrophages. Under the stimulation of chemotaxis (CCL2), the fusion-competent macrophages increase cell motility, form filopodia, and express cell fusogens (a molecule that fuses biological membrane, CD44, CD47/SIRPα, CD200/CD220R, DC-STAMP, CD36, E-cadherin) for cell-cell interactions^7–11^. These cells adhere to each other and rearrange their cytoskeletons (F-actin) via integrins for fusion^12,13^. Finally, the arrangement of cytoskeleton regulates the interchanges of intracellular and membrane content, and leads to multinucleation^14^. This macrophage fusion model implied that the macrophage fusion competency requires chemical fusogenic stimuli such as cytokines to regulate integrins expression during the process. However, integrins also function as mechanical sensor to respond to the physical property of biomaterial^15^. Hence, this chemicals-induced macrophage fusion model is not sufficient to explain biomaterials-induced macrophage fusion.

Although implanted-biomaterials are the main cause of MNCs formation during the foreign body reaction, the mechanism of the biomaterial-induced fusogenic stimuli remains unclear. Many researchers consider cytokines from macrophages or chemical properties from biomaterials as the major source of fusogenic stimuli. Therefore, current *in vitro* models for studying the interactions between macrophages and biomaterials were mandatorily applied cytokines such as IL-4, IL-3, INF-γ and RANKL to induce MNCs^16,17^. Even though studies were attempted to emphasize the alteration of biomaterial mechanical properties, cytokines were still included in the culture medium to promote the macrophage fusion. Cytokines masked the impact of biomaterial properties, and hence biomaterial on macrophage fusion was seldomly addressed, especially in the *in vitro* model. Previous *in vitro* studies have showed that some biomaterials such as poly(ethylene terephalate) (PET) and agarose alone were capable to induce macrophage fusion^18–20^. In addition, biomaterial physical properties could alter macrophage activations and phagocytosis^21,22^. These work lead us to conjecture that biomaterial-specific fusogenic stimuli may be able to promote alternative fusion mechanism that is different from three typical cytokines derived models.

To investigate the biomaterial-derived macrophage fusion, we established a 3D cell Col-Tgel (collagen-based) culture model with tunable mechanical properties^23^. It is biocompatible to provide natural cell adhesion sites and transparent to observe cell activities directly under optical microscopes^23^. Moreover, the 3D matrix condition can alter the proliferation rate of murine myoblasts and human cancer cells^24,25^. Thus, we used this model to examine how the stiffness of collagen biomaterial to alter the cell proliferation and competency of macrophage fusion.

## Results

### The embedded Raw264.7 cell proliferation, cluster formation, and mobility were 3D matrices dependent

**Figure 1:**
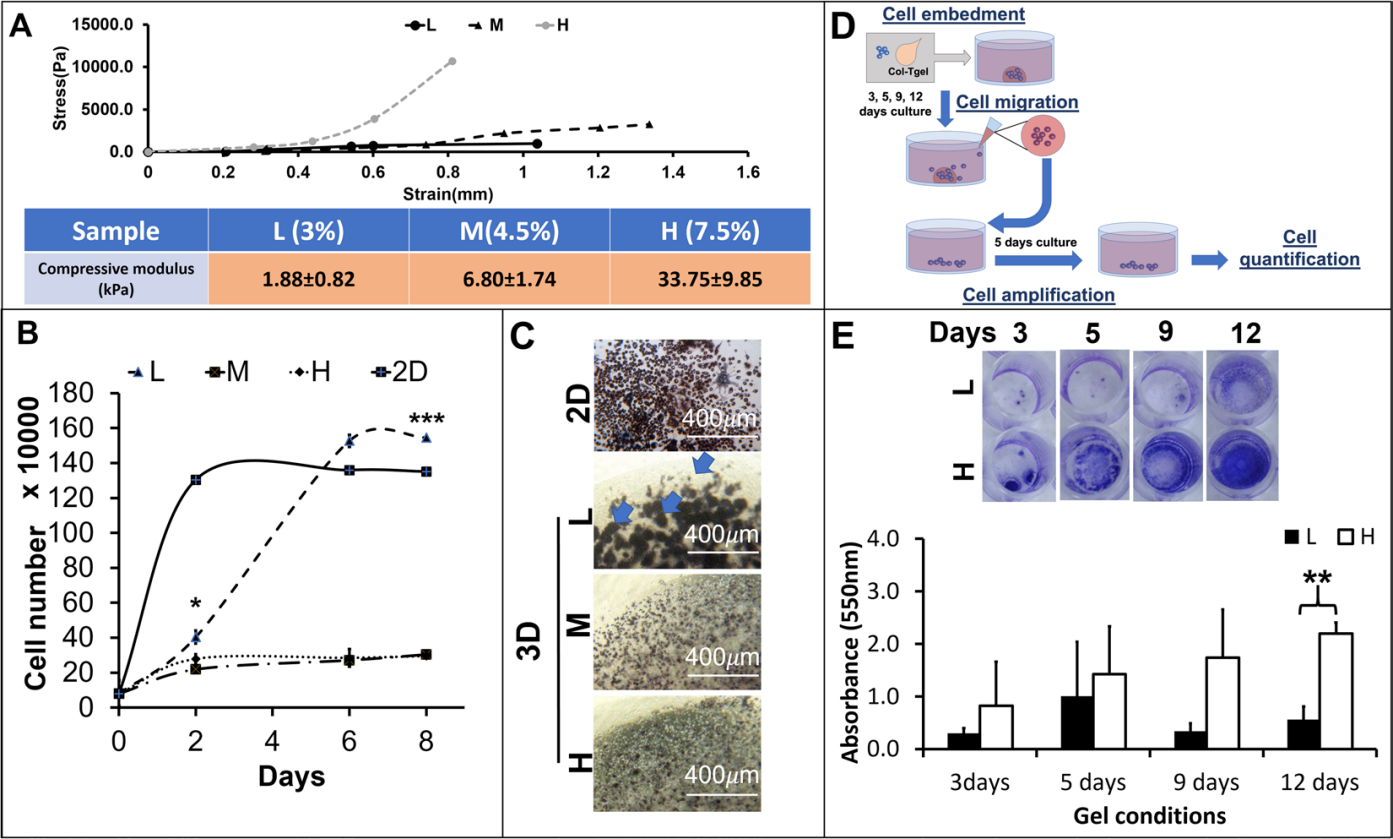
Alteration of Raw264.7 cells proliferation, cluster formation, and mobility by the 3D matrices. (**A**) The 3D matrices in different concentrations were measured their compressive modules by the unconfined compression test (n = 6, average ± standard deviation, (**B**). The scatter plot of cell proliferation in 3D matrices. The proliferation rates were calculated by counting the cell number from the Raw264.7 embedded in different 3D matrices, n = 3, 2^nd^ day: p = 0.1251, 6^th^ day: *p < 0.05, 8^th^ day: ***p < 0.001); (**C**) The cluster formation patterns. Optic images on the Raw264.7 cells embedded in different 3D matrices with MTT staining (blue arrows). (**D**) A diagram to illustrate the proposed experimental procedure for quantification of the migrated cells. (**E**) The absorbance measurement of the migrated Raw264.7 cells from the 3D matrices to culture medium, crystal violet staining at 3, 5, 9, and 12 days, n = 3, **p < 0.01.

To investigate the 3D-matrix effect on the embedded macrophages, three gel concentrations (3, 4.5 and 7.5%) were selected to generate different gel rigidities. Based on gel concentrations, the 3D matrices were defined as L (3%), M (4.5%), and H (7.5%). The 3D matrix displayed various stress-strain profiles depending on gel concentration. A 1.5-fold and 2.5-fold increase of gel concentration led to the 2-fold and 14-fold increase in the mean of the compression modulus respectively (Fig. 1A).

The Raw264.7 cells in the different culture conditions showed distinctive growth patterns (Fig. 1B). The Raw264.7 cells in the 2D culture (initial culture density: 80000cells/0.98 cm^2^) displayed the shortest lag phase (<48 hours) and followed by the L matrix condition (48 hours), but lacked lag phase in the M and H matrices. Coherently, The Raw264.7 cells in the 2D culture also presented the shortest doubling time (11 hours) than the 3D matrices, and followed by the L-matrix (20 hours), the M-matrix (66 hours) and the H-matrix (69 hours).The Raw264.7 cells in the 2D showed the shortest time taken to reach the maximum cell number and followed by the L matrix (6 days). The cell number in M and H matrices did not even reach their maximum during the experimental time frame. Their total cell number only increased approximately 3 folds after 8 days.

Based on the previous growth curves, the 3D matrices induced two types of growth rates. Consistently, the result of cell viability test (MTT assay) was also shown two divergent MTT intensity and cell clusters population on Raw264.7 cells in the different culture conditions. The cells in the 2D culture and L matrix showed the higher MTT intensity than the cells in the M and H matrices. These MTT stained cells formed cell clusters in all types of 3D matrices (Fig. 1C, blue arrows), and presented bigger (~150 µm in diameter) and more abundant in the L matrix than those in the M and H matrices (~50 µm in diameter).

Figure 1D illustrates an experimental procedure to quantify the Raw264.7 cells mobilities in different 3D matrices. The dye intensity (absorbance) in the H matrix was higher than in the L matrix (Fig. 1E). These differences increased as extending culture time and became the statistically significant on day 12 (p < 0.01). Higher dye intensity reflected a higher migrated cell population in the H matrix and also indicated that the Raw264.7 cells were highly motile in the H matrix.

Taken together, these results suggest that the mechanical properties (compressive moduli) could alter the cell proliferation, the cluster formation, and the motility of the Raw264.7 cells. These high proliferative cells resulted in high cell cluster population. Low proliferative led to fewer cluster populations, but higher motility.

### The 3D matrix-induced Raw264.7 cell fusion from the cell clusters without cytokines

**Figure 2:**
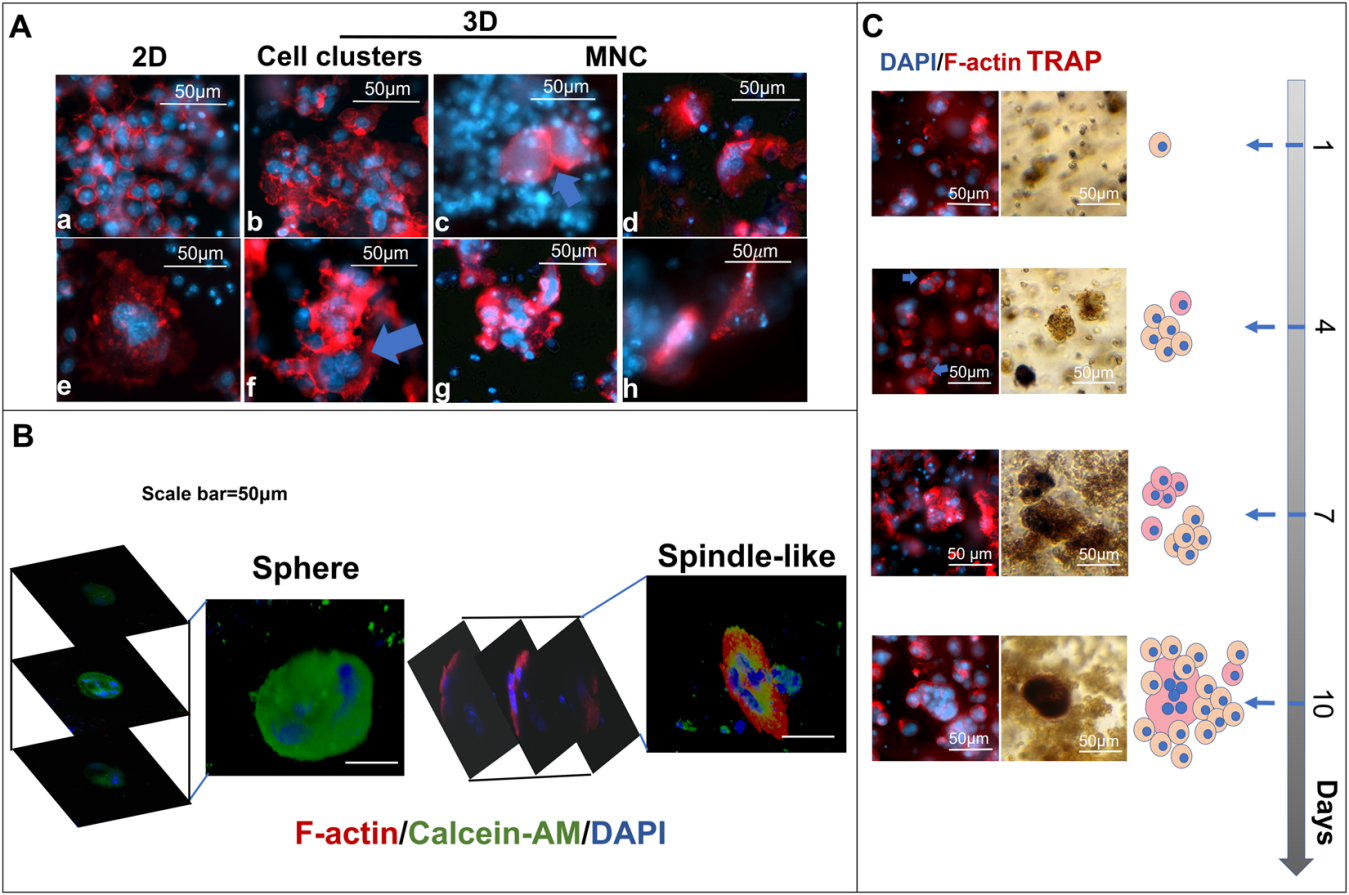
Morphological alteration of Raw264.7 cell clusters and fused cells by the 3D matrix. (**A**) The morphology of Raw264.7 cell-derived clusters and fused cells in 2D and 3D cultures (3%: b, f, c, g, and 4.5%: d and h)., F-actin (red, rhodamine phalloidin) and nuclei (blue, DAPI). (**B**) The 3D constructive images from the z-axis scanning of fluorescence confocal microscope. (**C**) A series of sequential images obtained from the 3D matrix (3%) embedded Raw264.7 cells cultured for 1,4,7, and 10 days. TRAP(dark red), F-actin (red, rhodamine phalloidin) and nuclei (blue, DAPI).

Based on the cell viability test, cell cluster sizes and numbers were altered in the matrix-dependent manner. To investigate whether the fusion occurs within these 3D matrix-induce cell clusters, the nuclei and cell membrane were detected by DAPI (DNA, blue) and rhodamine phalloidin (F-actin, red) to distinguish cell morphology and nuclei number within a cell. The 2D clusters (Fig. 2A-a) showed organized and equal-dense F-actins between each cell. Contrarily, the 3D clusters showed irregular and overlapped F-actins between cells (Fig. 2A-b). The cell-cell interactions of the 2D clusters restrained on the x-y panel, whereas the 3D clusters extended their cell alignment on the x-z, and y-z panels.

The 3D MNCs appeared either as part of cell clusters (Fig. 2A-c,f) or by themselves (Fig. 2A-d,g,h) and their shape were impacted by the 3D-matrix. Unlike 2D MNCspreading its cytoskeleton on the x-y panel, the 3D-matrix-oriented MNCs displayed three types of shapes: spherical, amoeboid-like, and spindle-like (Fig. 2A). The spherical shape was the most common MNC shape observed in all gel conditions (Fig. 2A-c,d,f). The amoeboid-like MNC only appeared in the L and M matrices. This particular MNC had filopodia, that seemed to phagocytose its neighbors to achieve cell fusion (Fig. 2A-g). The spindle-like MNC appeared only in the M and H matrices (Fig. 2A-h). Unlike spherical MNC, which was round in shape when observed from any of the 3 axes (Fig. 2B), the spindle-like MNC looked like spindle from the z-axis, but looked round in shape when viewed from x or y-axes. Due to these variations, it was renamed to the compressed-disc MNC.

The 3D matrix effect on Raw264.7 cell morphology and macrophage activation takes time and requires actin arrangement. Figure 2C showed a time course of cluster formation and cell fusion from the 3D matrix-embedded Raw264.7 cells. The mononuclear cells in either proliferative (L matrix) or motile (H matrix) competent matrix could aggregate into cell clusters (after 2–4 days). The cell clusters started fusing after 4 days. The fusing cells displayed accumulation of punctate actin (podosomes) and formation of filopodia (Fig. 2C, blue arrows in the left column). With extended culture time (day 10), more nuclei (4 nuclei) per fused cell could be observed. Additionally, the TRAP expression (Fig. 2A, right column), an indicator for active macrophages, occurred simultaneously with the formation of cell clusters. It could express partially in the cell clusters on the day 7 and fully in a fused cell on day 10.

These observations suggested the macrophage fusion and morphologies of 3D-matrix-derived MNCs were matrix dependent. The 3D matrix constrained the F-actin organization and the cell-cell interaction to determine the shape of cell clusters as well as the MNCs morphologies in the 3D matrices.

### The 3D matrix induced cell-matrix interaction diverted the cell fusion-related protein gene expressions

**Figure 3:**
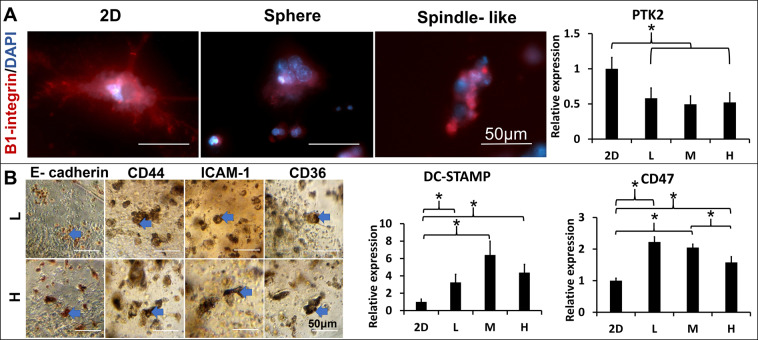
Localization of the cell-matrix and cell-cell adhesions in the 3D matrices-restrained Raw264.7 cells. (**A**) The cell-matrix adhesion of Raw264.7 cells derived MNCs in the L or H matrices was identified by the β1-integrin(red) expression with fluorescence immunocytochemistry. (**Left**); The integrin-mediated focal adhesion gene (PTK2) expression from the 2D and 3D (L, M and H matrices) cultured Raw264.7 cells was quantified by qPCR (n = 3, *p < 0.05) (Right). (**B**) The cell-cell interactions were identified by stainings of the E-cadherin, CD44, ICAM-1, and CD36  (brown) with peroxidase immunocytochemistry in the L and H matrices. (**left**). The macrophage fusion competency was quantified by the expression of cells fusion protein genes (DC-STAMP and CD47) from the 2D and 3D (L, M and H matrices) cultured Raw264.7 (**right**), *p < 0.05.

Morphology diversity led us to speculate that the embedded cells might sense physical signals and express adhesion sites accordingly. Cell adhesions include cell-matrix and cell-cell. The cell-matrix induced integrins mediate focal adhesion and trigger intracellular signals to promote macrophage fusion^13^. The β1-integrins, a member of transmembrane adhesion receptors that specialized bind to collagen, was used to identify cell adhesion sites on the cell membrane^26^. The spherical MNCs, similar to the 2D MNCs, presented the extended and scattered β1-integrin on the cell surface, but the compressed-disc MNCs presented condensed and clustered β1-integrin on one side of the cell surface (Fig. 3A). Focal adhesion kinase (FAK, gene name PTK2) is the central node of the signaling network emanating from focal adhesion. The PTK2 gene downregulated in the 3D matrix embedded cell than cells in the 2D culture, but the gene expression of PTK2 had no differences among different 3D matrices. The result indicates PTK2 related focal adhesion might have no difference in total embedded cells but β1-integrin expression varies between cells in different morphologies.

To localize the cell-cell adhesions, E-cadherin, CD44, ICAM-1, and CD36 were used to identify where the fusion event occurred. IHC showed that these proteins located in the cell clusters and either expressed partially or completely on the cell cluster (Fig. 3B, blue arrows). The overall proteins expressions of CD44, ICAM-1, and CD36 were decreased and restricted to the margin as increasing the matrix concentration (Supplement Fig. 1). Moreover, the 3D matrix condition also impacted macrophage fusion competency via the gene expression of dual function proteins such as DC-STAMP and CD47 on cell-cell adhesion and fusion. The gene expression of DC-STAMP and CD47 were both altered by the 3D matrix conditions, but their trends were opposite in accordance to their matrix stiffness.

Altogether, both cell-matrix and cell-cell interactions could vary either on each cell or a whole cell graft. The 3D induced cell-matrix adhesion relied on the cell morphologies, whereas fusion-associated cell-cell adhesion was matrix stiffness dependent. The macrophage fusion competency was altered by the 3D matrixvia alteration of fusion-associated cell-cell adhesion genes expressions.

### The macrophage fusion could be altered by cell-matrix adhesions

**Figure 4:**
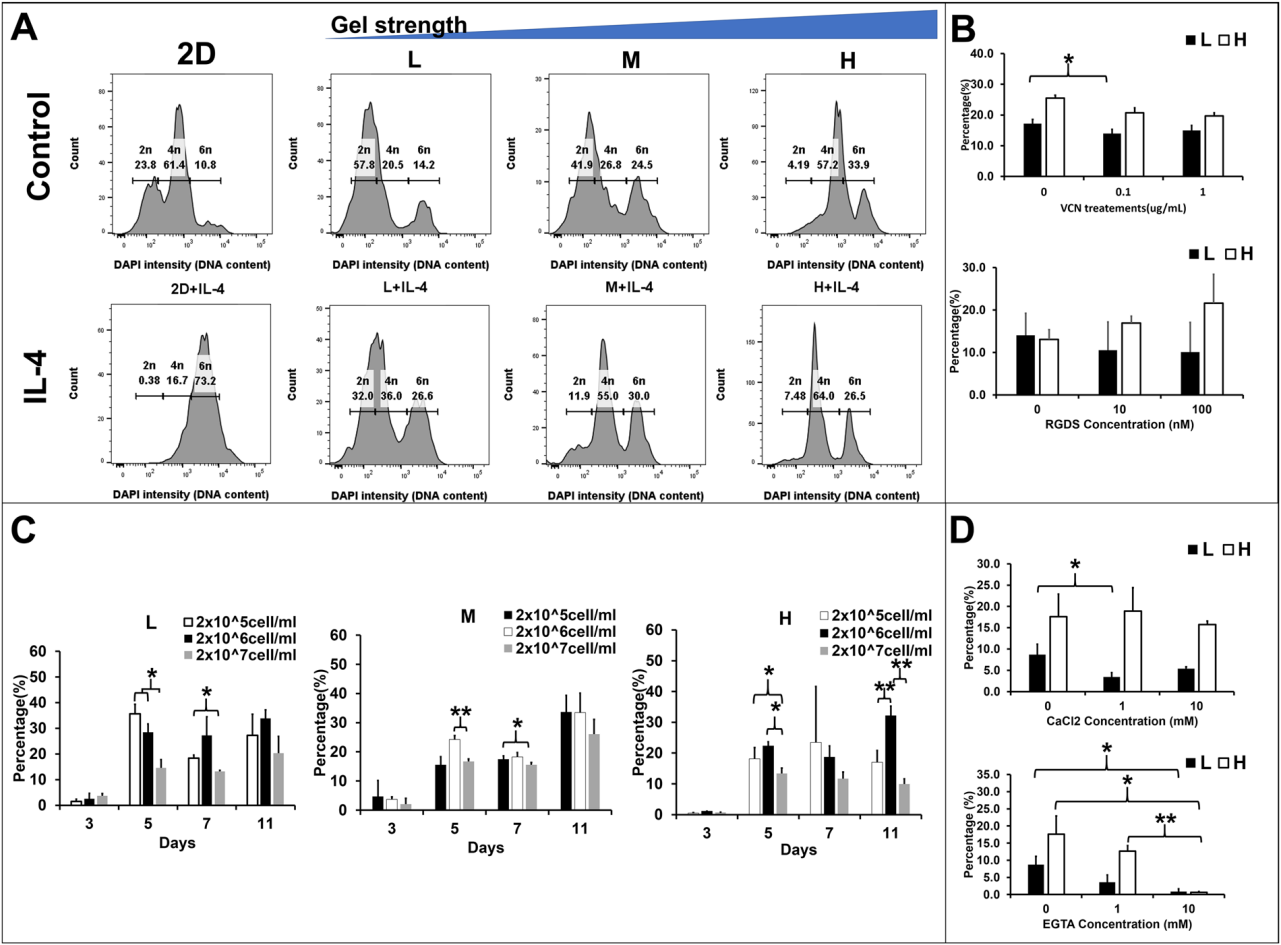
Alteration of cell densities or intervention of cytokines and inhibitors on the 3D matrix-induced cell fusion ratio via the cell-matrix and cell-cell adhesions in the Raw264.7 cells. (**A**) DAPI intensity (DNA content) of 2D and 3D (L, M, and H matrices) cultured Raw264.7 cells with/without IL-4 (20 ng/mL) from FACS assay. The DAPI density  corresponding to single nucleus (2n), double nuclei (4n), and triple nuclei (6n) respectively. (**B**) The bar graphs exhibited the percentage of fused Raw264.7 cells under different doses (0, 0.1, and 1) of cell-matrix adhesion inhibitors (VCN and RGDS) in the different matrices (L: black and H: white) for 7 days, *p < 0.05. (**C**) The Raw264.7 cells fusion rates were calculated by the fused cell (>3 nuclei) number over total cell number from different Raw264.7 cell densities (2 × 10^5^: white, 2 × 10^6^: black, 2 × 10^7^: gray cells/ml) embedded in the 3D culture (L, M, and H matrices). (**D**) The Raw 264.7 cell fusion rates with supplemetation of CaCl2 or calcium chelator EGTA.

The alteration of macrophage fusion competency led us to suspect that the matrix-induced fusogen could alter the cell fusion rate via cell adhesions. Two types of methods were designed to test whether the increase of cell-matrix adhesions by the matrix concentration or the integrins expression could increase the fusion rate. To quantify the fused cell nuclei number and population, the DAPI, which bound to the DNA, displayed different intensity according to the DNA content in the cell. Histograms presented three peaks that represented of single nucleus (2n), double nuclei (4n), and triple nuclei (6n) respectively from the DNA content of the Raw264.7 cells (Fig. 4A). The L and M matrices contained higher population of single and double nuclei compared to the H matrix, whereas the H matrix contained highest population of double and triple nuclei (Fig. 4A) among the three matrices. The Raw264.7 cells demonstrated the highest fused cell (6n) ratio in the H matrix (34.43%, Supplement Fig2. 6n, Control). However, the DNA content higher than 6n that was observed in the previous experiments (Fig. 2C,spindle-like MNC and 2D-spherical MNC) could not be detected by FACS.

IL-4, a potent lymphocytes-derived fusogenic stimulus that is commonly used to induce macrophage fusion, could increase the expression of β2-integrin on monocytes^27^. Hence, IL-4 was applied to examine whether cytokine-induced cell-matrix adhesion could enhance macrophage fusion. The histograms showed that high DAPI intensity peaks increased in all 3D matrices after IL-4 treatment (Fig. 4A). The percentage of cell population increased the 4n cells in all 3D matrices (L: 21.2 to 34.2%, M: 27.77 to 54.9%, and H: 56.77 to 65.5%) and 6n cells in the L (14.6 to 25.77%) and M (24.67 to 30.17%) matrices after IL-4 treatment (Supplement Fig. 2). However, the percentage of 6n cells in the H matrix decreased (34.43 to 26.4%). These results suggest that cell fusion rate could be enhanced by increase gel concentration and IL-4 treatment, but the fusion enhancement effect by IL-4 could be matrix type dependent.

To inhibit cell-matrix adhesion, two integrin inhibitors vicrostatin (disintegrin, VCN) and RGDS were applied to intervene cell bindings. VCN bound to integrins αvβ3, αvβ5, and α5β1, and RGDS (a fragment of fibronectin) bound to αIIβ3, αvβ3, αvβ6, α5β1, and α3β1 integrins on the cell membrane^28,29^. VCN demonstrated inhibitory effect on both matrix conditions in a dose dependent manner, but cells were more sensitive to VCN in the H matrix than in the L matrix (Fig. 4B). RGDS showed opposite effects between the L and H matrices. The inhibitory effect was shown when cells were embedded in the L matrix, whereas the enhancement effect was shown in the H matrix. Despite lack of statistical significance, single integrin-targeted inhibitors might not sufficient to inhibit macrophage fusion. These results suggested that the macrophage fusion rate could be altered by the cell-matrix adhesions.

### The macrophage fusion could also be altered by cell-cell adhesions

To investigate the increase of cell-cell contacts on macrophage fusion, three cell seeding densities in the 3D matrices were selected. In the 2D system, the optimal density for macrophage fusion is >1 × 10^5^ cell/well in 96-well-culture plate^30^. As the Raw264.7 cells proliferate relatively slower in the 3D than in the 2D, a range of cell density from 2 × 10^5^ to 2 × 10^7^ cell/ml was chosen. In the L matrix, the Raw264.7 cells showed the least fused cells population when cell density was 2 × 10^7^ cells/ml (Fig. 4C). The percentage of fused cells showed no difference when cell densities were 2 × 10^5^ and 2 × 10^6^ cells/ml. In the M and H matrices, the 2 × 10^6^ cells/ml condition was the optimal cell density to reach the highest percentage of fused cells. The other two cell densities could still reach the same percentage as 2 × 10^6^ cells/ml on day 11 in the M matrix, but they could not achieve the same percentage as 2 × 10^6^ cells/ml on day 11 in the H matrix. The H matrix might hinder the cell fusion process even though they had cell-cell interactions at the beginning of the cell culture. The low cell density 2 × 10^5^/ml were favorable for cell fusion in the L matrix (high proliferation condition), whereas 2 × 10^6^ cells/ml was favorable for cell fusion in the M and H matrix. The highest cell density inhibited cell fusion in all matrices. Although the high cell density increased the chances of cell-cell interactions initially, cells still require a proper adhesion environment to trigger the fusion machinery.

Calcium ions are critical for cadherins function and cell-cell junction formation^31^. Calcium chloride, as calcium ions provider, has been shown to enhance the cell-cell junctions in literature ^32^. However, increasing calcium chloride concentration in culture medium failed to enhance macrophage fusion in the L matrix. The percentage of fused Raw264.7 cells were even inhibited in the H matrix (Fig. 4D). To inhibit the cell-cell adhesions, EGTA, as calcium chelator, has been shown to intervene cell-cell adhesions and cell fusion in a dose-dependent manner ^33^. In 3D, the EGTA exhibited inhibitory effect on cell fusion in both matrix conditions. These results suggested that calcium ion was necessary in the process of the 3D matrix-induce macrophage fusion but it may not be sufficient to enhance it.

### The 3D matrix-induced fusogenic stimuli presented a similar impact on bone marrow-derived monocytes

**Figure 5:**
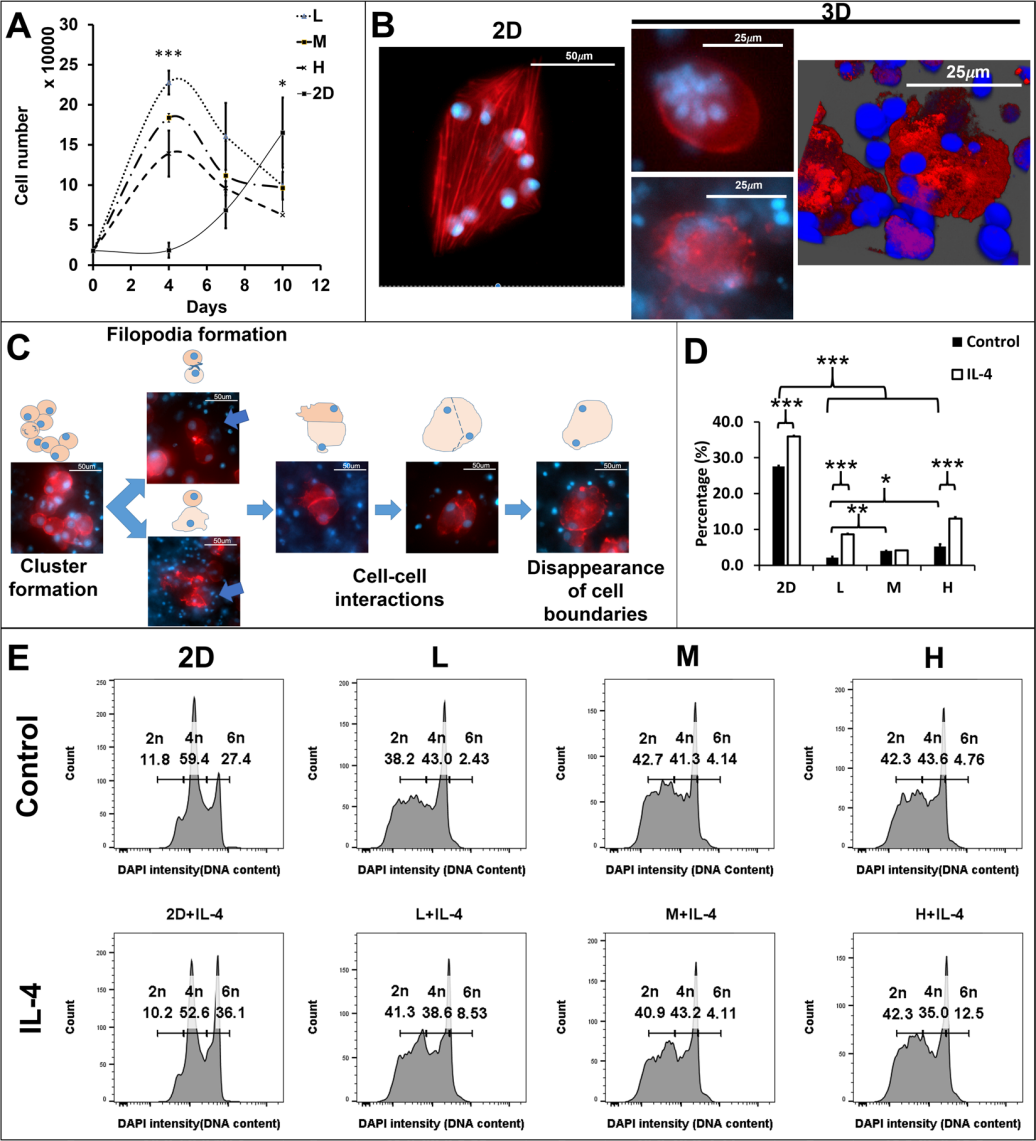
Modulation of rat bone marrow derived monocytes (BMDM) fusion by the 3D matrices. (**A**) The proliferation curves were derived from the 2D and 3D (L, M, and H) cultured BMDM for 0, 4, 7 10 days; (n = 3), 4^th^ day: ***p < 0.001, 7^th^ day: p = 0.093, 10^th^ day: *p < 0.05, (**B**) The fused BMDM morphologies were detected by F-actin (rhodamine phalloidin, red) and nuclei (DAPI, blue) staining in 2D and 3D culture (L matrix) at day10. (**C**) Flourescence images with schematic diagrams to describe BMDMs fusion process. (**D**) The fusion rate of BMDM in the 2D and 3D (L, M, and H matrices) cultures for 10 days (n = 3), ***p < 0.001, **p < 0.01, and *p < 0.05. (**E**) The histograms of the DAPI intensity (DNA content) of the 2D and 3D (L, M, and H matrices) cultured BMDM for 10 days.

To validate the observations in the Raw264.7 cells model, the rat bone marrow-derived monocytes (BMDM) were used to embed in the same 3D matrices. The BMDM cells showed no lag phase in the 3D matrix, but the lag phase in the 2D was 4 days (Fig. 5A). The cell doubling time from 2D, L, M, and H matrices were 46, 27, 29, and 33 hours respectively. The time taken to reach the maximum cell number was the same among 3D matrices (4 days), but the 2D culture took longer to reach the maximum cell number (>10 days). The cell states in the 3D matrix could be defined as high, medium, and low proliferative from L to H matrix.

Additionally, the BMDM fusion could also be observed in the 3D matrix, with up to 15 nuclei per cell in the 3D matrix (Fig. 5B). The nuclei distributed either in the center or at periphery. The actin distribution showed no difference between these two types. Morphologically, BMDM commonly formed the compressed-disc like MNC in the 3D matrix.

Similar to the Raw264.7 cells, the BMDM fusion process included the formation of cell clusters, the formation of filopodia (Fig. 5C, blue arrows), cell-cell adhesions, and disappearance of cell-cell boundaries. The BMDM fusion rate was quantified by the population of fused cells with triple nuclei (6n) or more. The BMDM fusion rates were higher in the 2D culture than in 3D matrices (Fig. 5D,E). In the 3D matrix, the H matrix demonstrated the highest fusion rate (5.2 ± 0.66%, Fig. 5D). The cell fusion rate increased in all 3D matrices after IL-4 treatment.

Compared to the Raw264.7 cells, BMDM showed more uniform in morphology and larger number of nucleus in the fused cells.

### The 3D matrix altered the multinucleated cells population in the *in vivo* model

**Figure 6:**
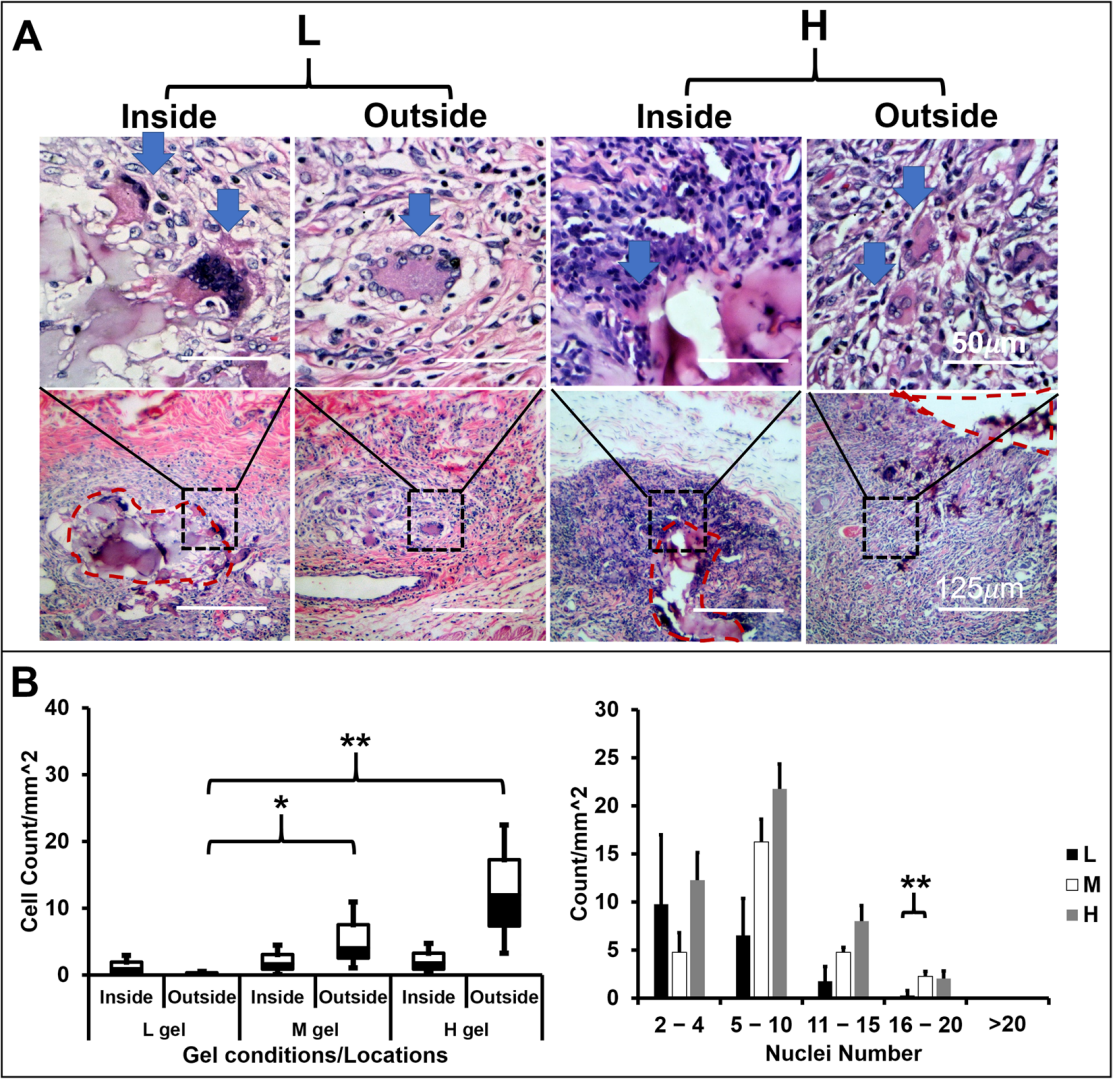
Alteration of MNC population by the 3D matrices in the *in vivo* model. (**A**) The morphologies and nuclei distribution of MNCs. Histographs genearted from parafilm-embedded microsections of the 3D gels (L, M, and H) implanted in Fisher344 rat for 7 days. H&E staining. (**B**) The MNCs densities inside or outside of gels were computed by the MNCs number/mm^2^ from the explants, (n = 4, **p < 0.01 and *p < 0.05).

Finally, the 3D matrices were intramuscularly implanted in animals to investigate MNC formation, morphology and population. Two types of MNCs that their nuclei either center or periphery concentrated, could be observed in the 7-day explants. The nuclei centered MNCs were usually observed inside the gel, whereas the nuclei periphery concentrated MNCs were observed outside the gel (Fig. 6A). The MNCs number outside the 3D matrices increased as increasing the matrix stiffness (Fig. 6B). Contrarily, the MNC within the 3D matrices had no differences in number. The higher nucleus number of MNCs were observed in both M and H matrices, especially when nuclei number ranging from 16–20. The *in vivo* models demonstrated a similar morphology and the trend of the population as increasing the matrix stiffness as *in vitro* BMDM model.

## Discussion

This study aims to address whether the collagen-based 3D matrices can impact macrophage fusion. The main findings are that the mechanical property of the collagen-based 3D matrix, also named as 3D matrix-induced fusogenic stimuli, can regulate macrophage fusion competency and fusion rate in the Raw264.7 cell and the rat BMDM. Our results demonstrate that the 3D matrix-induced fusogenic stimuli alter the cell state of macrophage in three aspects: 1) the cytoskeleton (F-actins, Fig. 2B), 2) the cell-matrix adhesion proteins (β1-integrins) and the cell-cell adhesion proteins (Fig. 3A,B, and Supplement Fig. 1), and 3) the cell fusion protein genes (CD36, CD44, DC-STAMP and CD47, Supplement Fig. 1 and Fig. 3B). The alteration of these parameters modulates macrophage fusion (Fig. 4).

These 3D matrix-induced fusogenic stimuli present bifunctional properties including the chemical binding sites from collagen and the physical restrictions from the 3D matrix. Unlike the general chemical stimuli that can diffuse freely and impact cells systematically, the 3D matrix-induced stimuli are stable on the material interface that impact cells regionally. It is evidenced that the distribution of F-actin and β1-integrin changed as the matrix stiffness increased(Fig. 2B). Similar observations have reported by Féréol *et al*. that the morphology of alveolar macrophages changed from round to flattened as substrate stiffness increased^34^. It also coherent with findings from Padmanabhan *et al*. that bone marrow-derived macrophages extended or elongated their cytoskeletons depending on the pattern of the material surface^35^.

Despite the regional effect of 3D matrix fusogenic stimuli, the overall macrophage fusion competency impacts the percentage of fused cells. Both cell fusion protein expressions (CD44, CD36, CD47 and DC-STAMP, Supplement Fig. 1 and Fig. 3B) and the percentage of fused cells were altered responding to the stiffness of the 3D matrix in the Raw264.7 cells (Fig. 4A and Supplement Fig. 2). The alteration of the fused cell population was also observed in the primary BMDM, and Fisher 334 rat model (Figs. 5F and 6B). These evidences support our hypothesis that the stiffness of the 3D matrix altered the fusogen on macrophage to control the quantity of fused macrophage.

**Figure 7:**
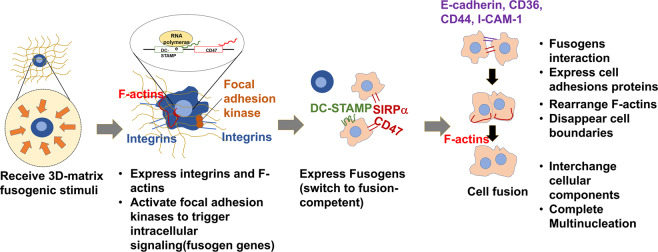
A schematic illustration of biomaterial-induced macrophage fusion. A proposed mechanism includes four steps: (1) receiving 3D-matrix stimuli, (2) forming cell-matrix adhesions, (3) expressing fusion-associated cell-cell adhesions, and (4) rearranging cytoskeletons and interchanging cellular content.

Our findings suggest a new model (Fig. 7) for the macrophage fusion mechanism. This model begins with the biomaterial-induced fusogenic stimuli and macrophages interactions. Macrophages express integrins to adhere to the biomaterial substrate and F-actins to respond to the solid stress from biomaterial. These integrins and F-actins trigger focal adhesion signaling cascade to activate cell fusion protein genes^36^that switche macrophages to fusion competent. The fusion competent macrophages adhere to each other and form cell-cell adhesions. Finally, their F-actins rearrange to interchange cellular components and complete multinucleation. In this model, the macrophages adhesion on biomaterial is a critical step to initiate biomaterial-induced macrophage fusion.

In contrast to previous reports, there are two major differences between our studies and others. First, we found the highest fusion rate did not occur in the high proliferation matrix condition. Most studies correlate high macrophage population to high macrophage fusion or foreign body reaction in both *in vitro* and *in vivo* models^37,38^. In our model, macrophages presented the highest fusion rate in the matrix condition that induced low proliferation, but high motility. This observation suggest that cell density is not sufficient to promote macrophage fusion, but cell motility is necessary for macrophage fusion. Second, the highest fusion rate did not occur in the matrix that induces the highest CD47 gene expression. Both CD47 and DC-STAMP are considered to correlate positively with macrophage fusion^39^. Ida *et al*. found the fusing Raw264.7 cells increased DC-STAMP expression as decreasing the matrix stiffness by lysyl oxidase (LOX) inhibitor (β-aminopropionitrile)^40^. The expression of CD47 on stiff substrate does not correlate to macrophage fusion, but Sosale *et al*. demonstrated that macrophages increased phagocytosis when sensed substrate stiffness via the expression of CD47^41^. In this study, the CD47 highly expressed in the matrix that was optimal to induce amoeboid-like MNCs (Figs. 2A-g, 3B). Xing *et al*. suggested that the fusion machinery might be regulated by different pairs of fusion proteins. They showed that CD47 expression could be reduced while DC-STAMP expression could be enhanced after the RANKL treatment^42^.

Interestingly, we found two unexpected results in our model. First, the morphologies of primary BMDM derived MNCs were similar to MNCs *in vivo* model (Figs. 5B and 6A). These MNC morphologies are also similar to the cytokines-induced MNCs from the primary human blood monocyte model performed by McNally and Anderson. They found that the nuclei arrangement of MNCs were different (peripheral and center-concentrated) depending on the combination of cytokines^43^. These two types of MNCs correlate to Langhan giant cells and biomaterial induced foreign body giant cells in the clinical manifestations^44^. This 3D model may be relevant to study the foreign body reaction *in vitro*. Second, the IL-4 effect on the fusion rate of macrophages is 3D matrix dependent. In the Raw264.7 cells model, IL-4 enhanced macrophage fusion only in the matrices that performed a low fusion rate. In the matrix that performed a high fusion rate, the material effect overrides the IL-4 effect. This observation is coherent with Jagannath *et al*. that primary biophysical cue from the nanotopography can override IL-4 effect on macrophage fusion. These results suggest that material can impact the macrophage sensitivity to chemicals, such as cytokines or drugs.

Nevertheless, there are three limitations to this model. First, the flow cytometry could not detect the fused cell containing more than 4 nuclei. To resolve this problem, the fluorescence image system can be used to identify the fused cell containing high nuclei and calculate these fused cells manually. Second, the host tissue contains other types of immune cells and stromal cells to intervene in the macrophage-biomaterial interactions. These cells are also impacted by the 3D matrix to compete the growth space, and produce cytokines or chemokines. To improve the model, including tissue-specific cell types can simulate a physiologically relevant condition in *in vitro*. Third, macrophages exhibit activation disparities among species, strains, and tissues. For example, the macrophage cell lines expressed less cytokines after chemical stimuli (lipopolysaccharide, LPS) treatment when compared to primary cells^45^. Primary macrophages derived from C57BL/6 and Balb/c showed Th1 and Th2-biased cytokine expressions^46^. Moreover, primary macrophages from the same strain but different tissue sources could also present different macrophage fusion rates^47^. Although the source of macrophage determines its activation tendency, these evidences still benefit us to understand the plasticity of MNCs to adapt microenvironment.

## Materials and methods

### 3D-matrix preparation

3D-matrices (Col-Tgel) were prepared as previously described^24,25^. The gelatin solution was diluted into 3%(L), 4.5%(M) and 7.5%(H) (v/v) with the phosphate buffered saline (PBS, 137 mM NaCl, 2.7 mM KCl, 10 mM Na_2_HPO_4_, and 1.8 mM KH_2_PO_4_). Microbial transglutaminase was purified from *Streptomyces mobaraense* (Ajinomoto) with SP Sepharose Fast Flow beads (Sigma-Aldrich) as described by Fang *et al*.^23^. The purified transglutaminases were stored at −80 °C before use. Gels were solidified at 37 °C for 1 hour and prepared for cell embedment and compression tests. All chemicals were purchased from Sigma-Aldrich.

The compressive modulus (E) was measured by the unconfined compression test that applied lateral deformation with parallel plates geometry as described by Tan *et al*.^24^. Samples were molded into a cylindrical shape, with height 20 mm and diameter 15 mm. The test was performed on six freshly prepared samples. The force (σ) and sample deformation (δL) were recorded. The strain(L*) is defined by δL/L, where L is the total height of the cylindrical sample. E given as E = σ/L* was computed from the slope of the stress-strain curve.

### Primary monocyte isolation

Bone marrow from 3month-old male Sprague-Dawley rats was isolated by flushing the femur with PBS containing 2% bovine serum albumin. Red blood cells were lysed (0.74%NH_4_Cl, 0.017%Tris-HCl) and removed by centrifugation. The remaining cells were rinsed in the RPMI medium followed by centrifugation. Cells were cultured for 7 days in the RPMI medium with a supplement of 10% fetal bovine serum and rat macrophage-colony-stimulating factors (5 ng/mL, M-CSF, Biolegend) before embedment.

### Cell embedment

The murine macrophage (Raw264.7, ATCC, VA) was cultured in high glucose Dulbecco’s modified Eagle medium (DMEM, Corning, VA). The primary bone marrow derived monocyte (BMDM) was cultured in RPMI-1640 medium with M-CSF. Both media were supplemented with 10% (v/v) fetal bovine serum (FBS, Hyclone, ThermoScientific) and 1% (v/v) penicillin-streptomycin (PS, Corning, VA). All cells were maintained in a humidified atmosphere (5% CO_2_, 37 °C) and the culture media were refreshed every 2-3 days. Cells were subcultured after the cell population reached 80% confluency.

Both types of macrophages were detached and dispersed evenly in the L, M and H matrices to 4 × 10^6^ cells/ml. A droplet with 20 µL of the cell-gel mixture was seeded on each well of 48-well suspension cell culture plate to form a half-dome shape on the well surface. After the gel solidified by enzymatic crosslinking (37 °C, 1 hour), 500 µL of cell type-specific medium was added into each well to submerge the grafts.

### Cell proliferation rate

To quantify cell growth, the 3D matrices-embedded macrophages were cultured in the medium for 2, 6, and 8 days and released by 0.25% trypsin-EDTA. The released cells were counted by particle counter (Z™ Series COULTER COUNTER® Cell, Beckman Coulter, CA). Single cells (gate range 9.49-20 μm)and large cells or cell clusters (>20 μm) were recorded.

### Cell viability assay

The cell viability was determined by the reduction of MTT(3-(4,5-dimethylthiazol-2-yl)-2,5-diphenyltetrazolium bromide)^48^. The cell grafts were rinsed twice with PBS and incubated with 500 µL of MTT working solution (5 mg/ml) at 37 °C. The cell grafts images were taken after 2 hours of color development under an inverted microscope (Leica, IL).

### Cell mobility test

To quantify the cell mobility, the proposed experimental procedure was illustrated in Fig. 1D.Both Raw264.7 cells and BMDM were embedded in L and H matrices and incubated for 3, 5, 9 and 12 days. The culture media together with migrated-out macrophages were collected on the predetermined timepoints and transferred into new tissue culture wells. the re-attached macrophages were refreshed every two days. After 5 days, the amplified macrophage were quantified by crystal violet assay^49^. Briefly, the adherent cells in the transferred wells were stained with crystal violet (0.5% w/v, Sigma Aldrich), and lysed with 100% ethanol to release the dye. The concentration of crystal violet was detected by the absorbance (550 nm) with multiplate reader (Molecular Devices, CA). Higher absorbance reflected a higher quantity of the migrated cells in the culture condition and referred to higher cell mobility condition, and vice versa.

### *In situ* immunocytochemistry

Tartrate-resistant acid phosphatase (TRAP) activity was tested by the *in situ* enzymatic histochemistry method according to manufacturer protocol (Cat. 387A-1KT, Sigma-Aldrich, MO).

For cell morphology, the 3D matrix-embedded macrophages were fixed with PBS buffered 10% formalin, and permeabilized with 0.05% Triton X-100. Their nuclei and cell membrane were labeled with DAPI dihydrochloride (1:10000, 15 min) and rhodamine phalloidin (F-actin, 1:1000, 30 min) respectively. To detect cell-matrix adhesion, rabbit anti-mouse β1-integrin primary antibody (CD29, 1:400) and Alexa 555 conjugated anti- rabbit secondary antibody (1:800) were applied on 3D matrix-embedded macrophages. All fluorescent images were obtained by a fluorescence microscope (EVOS, ThermoFisher Scientific, NY). Unless mentioned otherwise, all the fluorescence probes and antibodies were purchased from ThermoFisher Scientific.

For 3D images, cell grafts were cultured on the square cover slides (18 mm × 18 mm) for 10 days. The nuclei, cytoplasm, and cell membrane were detected by with DAPI, calcium-AM (1:1000), and rhodamine phalloidin respectively. The single or multiple (every 2.5 µm of increment in depth) fluorescent images were recorded and reconstructed with LAS X software under a confocal fluorescence microscope (Zeiss LSM 510 confocal microscopy imaging system, Leica, IL).

Immunolocalization of cell-cell adhesions were detected by rabbit anti-mouse or rat primary antibodies: E-cadherin (1:400, ThermoFisher Scientific, NY), CD44(1:400, ThermoFisher Scientific, NY), ICAM-1 (1:400, Bioss, MA), and CD36 (1:400, Bioss, MA). This was followed by the application of biotin conjugated goat anti-rabbit secondary antibody (1:800, Sigma-Aldrich, MO) and DAB-peroxidase substrate kit (ThermoFisher Scientific, NY).

### Analysis of *RNA* expression with qPCR

The RNA from different culture conditions(n = 3) were isolated by Quick-RNA Miniprep kit^®^ (Zymo Research, CA). The concentrations of the isolated RNAs were determined by the nanophotometer (Implen, CA). Primers were designed by PrimerQuest Tool^®^(Integrated DNA technologies, IA) for genes (Supplement Table 1). All transcripts were analyzed by the Bio-Rad CFX96 Touch^TM^ Real-Time PCR detection system (Bio-Rad, CA). The amounts of transcripts, relative to ACTB, were using the formula 2−∆∆Ct. The results calculated as relative expression to 2D culture.

### Fusion rate quantification with flow cytometry (FACS)

The cell grafts were cultured in the cell-specific medium for 7 days with/without mouse or rat IL-4 (Biolegend, CA) and then cultured in a serum reduced medium (1%FBS without M-CSF supplement) for 1 day to inhibit cell proliferation. Then, these 3D matrices-restrained macrophages were released with type-1 collagenase (Sigma-Aldrich, MO). The released macrophages were labeled with DAPI (1:2000) and detected by the LSRFortessa X20. Cell analyzer (BD Bioscience, NJ). The results were processed with FlowJo (BD Bioscience, NJ) and presented into histograms (count versus DAPI intensity).

### Fusion rate quantification with fused cell counting

To determine the fusion rate in the 3D-matrix, cell grafts were cultured for 7 days and then harvested to perform *in situ* immunocytochemistry with DAPI and rhodamine phalloidin as described in Method section 2.7. The fluorescence images (n = 10) were recorded and the fusion rate was computed as the number of fused cells (≥3 nuclei) in the 500 × 400 μm area.

### Implantation 3D-matrices in the *in vivo* model

The 3D-matrix in different conditions as described in the 3D matrix preparation was subcutaneously injected in three male Fisher344 rats (Charles River Laboratories, CA). Each 3D matrix condition had four injection sites. All animals were housed in USC Animal Resource Center and provided daily per diem. After 7 days of inoculation, cell grafts with surrounding tissue were explanted from the euthanized animals. The explants were embedded in paraffin and sectioned to perform H&E and immunohistochemistry staining. The number of MNC (≥3 nuclei) were calculated and normalized with cross-section area (mm^2^). All procedures were performed in accordance with Institutional guidelines and protocols that approved by University of Southern California Institutional Animal Use and Care Committee (USC IACUC).

### Statistical analysis

The significant differences between groups were analyzed with nonparametric ANOVA using SAS (SAS Institute Inc.). This analysis method was ideal for the small amount sample size. Tukey’s test was chosen for pairwise comparison as the sample size was the same. The difference was considered significant when *p < 0.05. Statistical bar graphs with mean and standard error of the mean (SEM)were plotted.

## Supplementary information

Supplement table and figure.
