# Supplementary material for: Switch of macrophage fusion competency by 3D matrices
Source: Sci Rep. 2020 Jun 25;10:10348. doi: 10.1038/s41598-020-67056-9 (PMC7316750; doi:10.1038/s41598-020-67056-9)
Supplement: Supplementary file 1 — Supplement table and figure. [file 41598_2020_67056_MOESM1_ESM.docx]

**Title:** Switch of macrophage fusion competency by 3D matrices

**Authors:** Josephine Y. Fang^1,2^, Zhi Yang^1^, and Bo Han^1^*

**Affiliations:**

^1^Nimni-Cordoba Tissue Engineering and Drug Discovery Laboratory, Division of Plastic and Reconstructive Surgery, Departments of Surgery and, Keck School of Medicine, University of Southern California, Los Angeles, California

^2^Center of Craniofacial Biology, Herman Ostrow School of Dentistry, University of Southern California, Los Angeles, California

**Corresponding Author:** Bo Han, Department of Surgery and, Keck School of Medicine, 1333 San Pablo Street, BMT-302, Los Angeles, CA 90089, United States. Phone: 323-442-2242; E-mail: bohan@usc.edu

**Supplement Data**


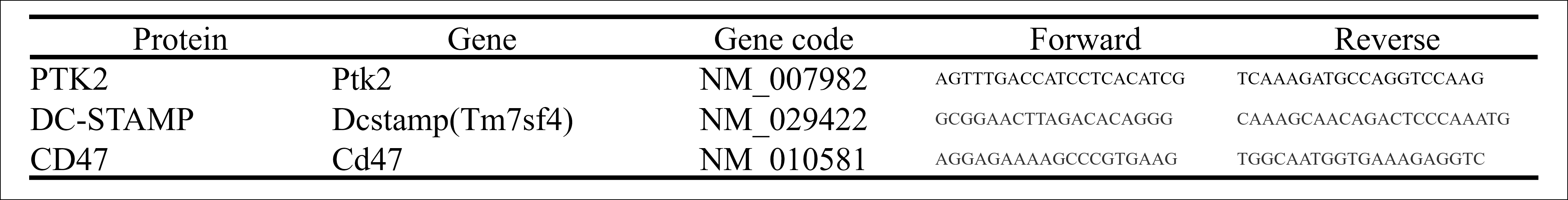


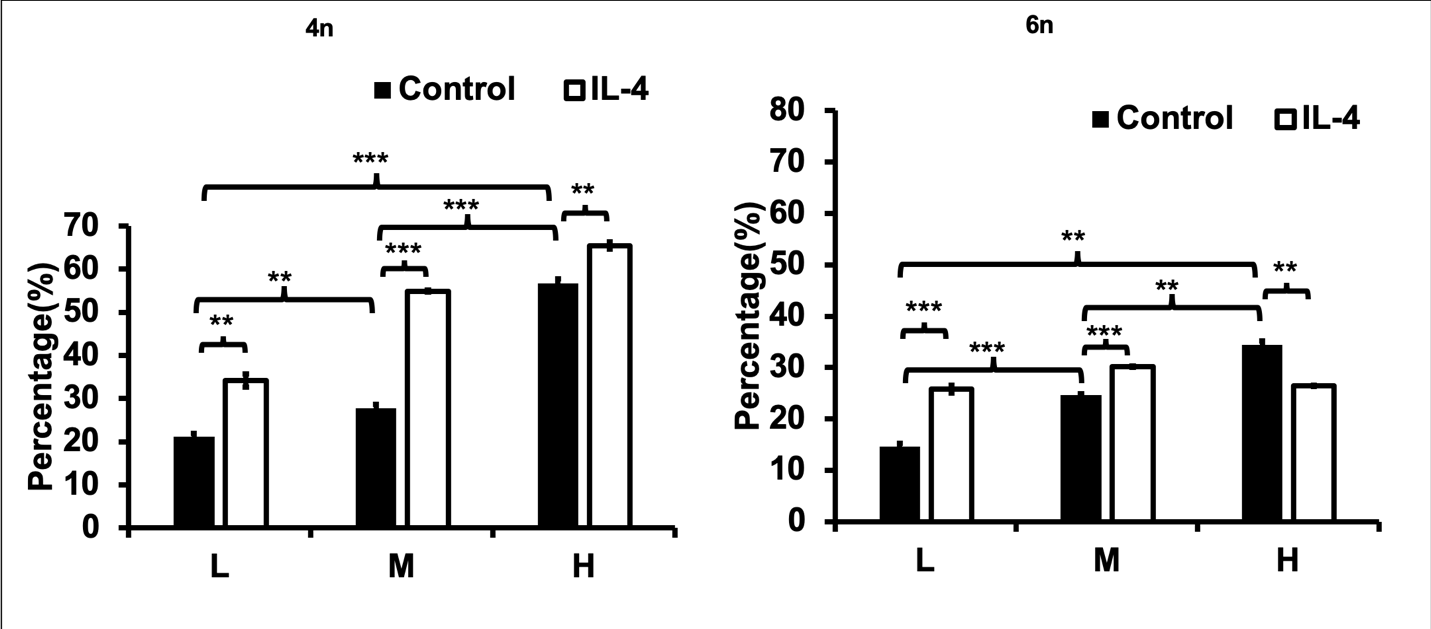


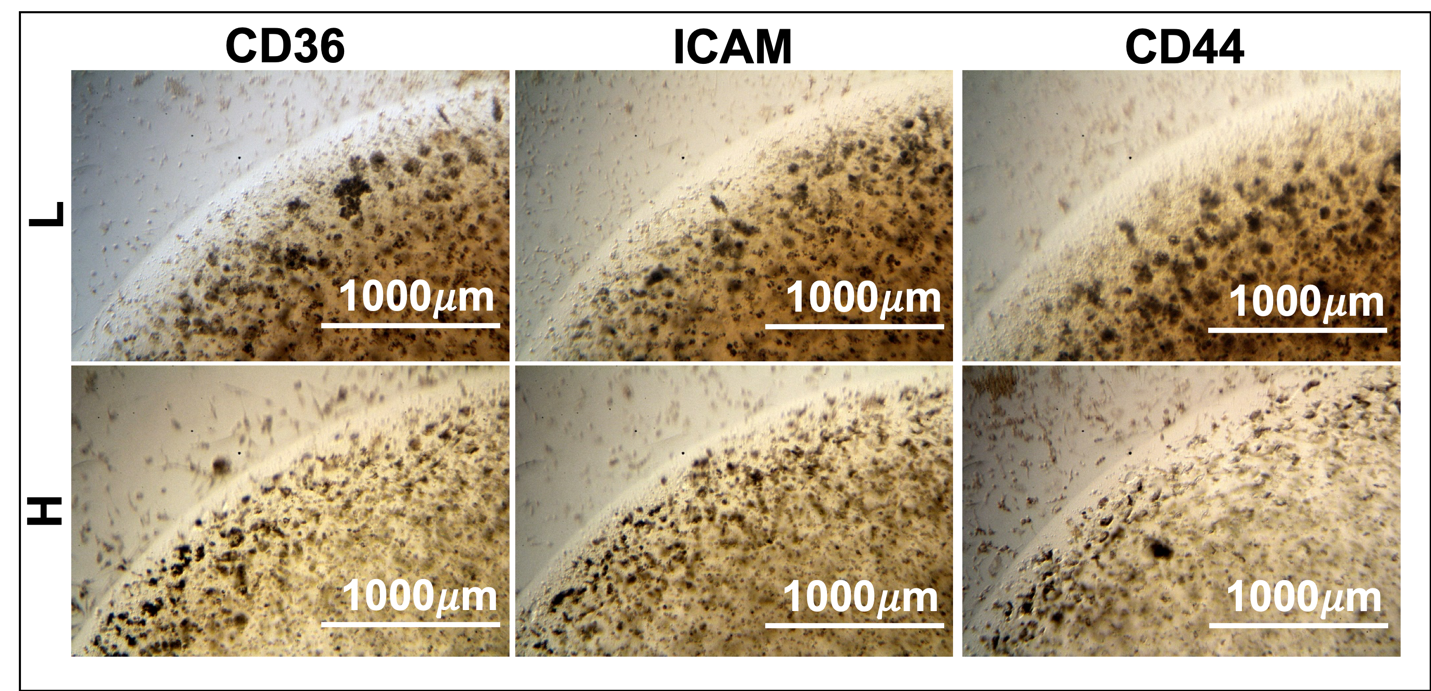


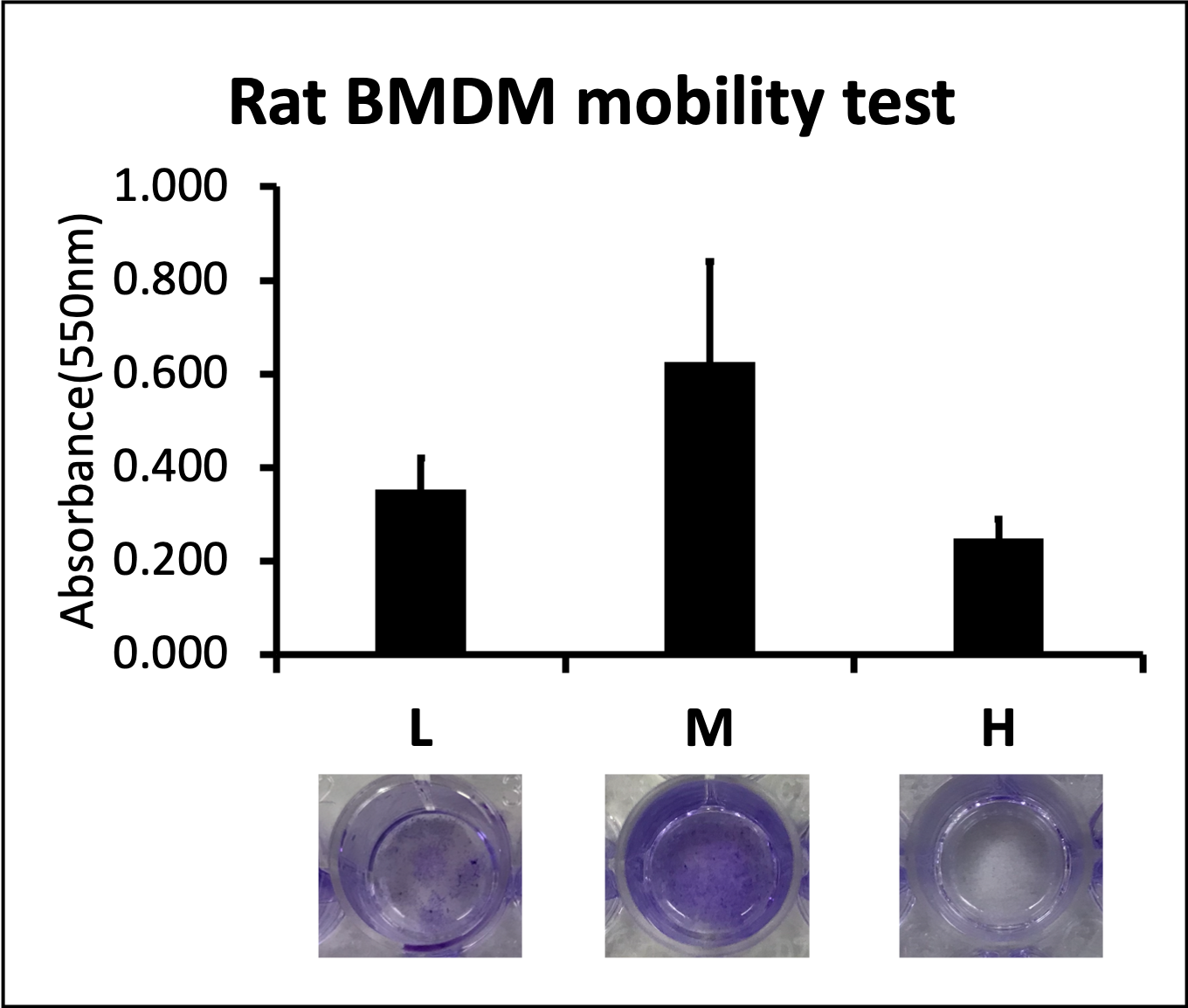


**Supplement data captions**

**Supplement table 1**. **Gene primers for quantitative PCR.**

**Supplement figure 1. Alteration of Raw264.7 cell-cell adhesion proteins by the 3D matrices.** Cell-cell adhesion proteins, CD36, CD44, and ICAM-1 were detected by the immunocytochemistry and visualized by DAB detection kit after 7 days culture.

**Supplement figure 2.** **Alteration of Raw264.7 cells fusion rate by the 3D matrices.** The bar graph demonstrated the fusion rate (4n and 6n) of 3D matrices-restrained Raw264.7 cells (4n or 6n cell number/total cell number x 100%) with/without IL-4 treatment (n=3,***p<0.001, **p<0.01).

**Supplement figure 3.** **Alteration of BMDM mobility by the 3D matrices.** The bar graph was BMDM mobility was quantified by the migrated Raw264.7 cells population from the 3D matrices. The migrated cells population were detected by crystal violate assay and measured the absorbance of cell lysate. Higher absorbance reflected higher migrated cells in the culture condition. The migrated cell population was quantified by the intensity of crystal violate staining.
